# Microelectromechanical Systems Based on Magnetic Polymer Films

**DOI:** 10.3390/mi13030351

**Published:** 2022-02-23

**Authors:** Denisa Ficai, Marin Gheorghe, Georgiana Dolete, Bogdan Mihailescu, Paul Svasta, Anton Ficai, Gabriel Constantinescu, Ecaterina Andronescu

**Affiliations:** 1 Department of Inorganic Chemistry, Physical Chemistry and Electrochemistry Faculty of Applied Chemistry and Materials Science, University Politehnica of Bucharest, Gh. Polizu 1-7, 011061 Bucharest, Romania; denisaficai@yahoo.ro; 2 National Research Center for Food Safety, University Politehnica of Bucharest, Splaiul Independentei 313, 060042 Bucharest, Romania; dolete.georgiana@gmail.com (G.D.); ecaterina.andronescu@upb.ro (E.A.); 3 National Center for Micro and Nanomaterials, University Politehnica of Bucharest, Splaiul Independentei 313, 060042 Bucharest, Romania; 4 Center for Technological Electronics and Interconnection Techniques, University Politehnica of Bucharest, Bulevardul Iuliu Maniu, 061071 Bucharest, Romania; maringhe@nanom-mems.com (M.G.); bogdan.mihailescu@elinclus.ro (B.M.); paul.svasta@cetti.ro (P.S.); 5 NANOM—MEMS, George Cosbuc 9, 505400 Rasnov, Romania; 6 Science and Engineering of Oxide Materials and Nanomaterials, Faculty of Applied Chemistry and Materials Science, University Politehnica of Bucharest, Gh. Polizu 1-7, 011061 Bucharest, Romania; 7 Academy of Romanian Scientists, Ilfov Street 3, 050044 Bucharest, Romania; 8 Department of Gastroenterology, Clinical Emergency Hospital of Bucharest, Carol Davila University of Medicine and Pharmacy, Bulevardul Eroii Sanitari 8, 050474 Bucharest, Romania; gabrielconstantinescu63@gmail.com

**Keywords:** magnetic polymer films, microelectromechanical systems–MEMS, design and fabrication, polymer selection

## Abstract

Microelectromechanical systems (MEMS) have been increasingly used worldwide in a wide range of applications, including high tech, energy, medicine or environmental applications. Magnetic polymer composite films have been used extensively in the development of the micropumps and valves, which are critical components of the microelectromechanical systems. Based on the literature survey, several polymers and magnetic micro and nanopowders can be identified and, depending on their nature, ratio, processing route and the design of the device, their performances can be tuned from simple valves and pumps to biomimetic devices, such as, for instance, hearth ventricles. In many such devices, polymer magnetic films are used, the disposal of the magnetic component being either embedded into the polymer or coated on the polymer. One or more actuation zones can be used and the flow rate can be mono-directional or bi-directional depending on the design. In this paper, we review the main advances in the development of these magnetic polymer films and derived MEMS: microvalve, micropump, micromixer, microsensor, drug delivery micro-systems, magnetic labeling and separation microsystems, etc. It is important to mention that these MEMS are continuously improving from the point of view of performances, energy consumption and actuation mechanism and a clear tendency in developing personalized treatment. Due to the improved energy efficiency of special materials, wearable devices are developed and be suitable for medical applications.

## 1. Introduction

Films based on polymer and magnetic materials are extensively used in industrial, and environmental, but also medical, applications [1]. The development of the magnetic films is especially beneficial because it can combine the advantages of the two components. Magnetic component is usually embedded into or coated onto the polymer film in order to confer to these films magnetic properties, hyperthermia capacity, monitoring capacity, etc., while the polymer assures some properties, including binder capacity for the magnetic powder, biocompatibility, release regulator capacity, stabilizing effect, especially in harsh conditions (acidic or chelating agents), etc. [2].

Metal oxides (such as Fe_3_O_4_, Fe_2_O_3_) are especially preferred to be used because they are more stable in high magnetization compared with the pure metals (such as Fe or Co) while the magnetic properties are suitable. Moreover, both magnetite and hematite can also be used in medical applications, being stable and biocompatible; the accidental release of the ions do not induce significant pH change and the level of the Fe^2+^/Fe^3+^ is in a safe range and does not induce inflammation or irritation. Moreover, it can be easily functionalized with adequate molecules and with passivation or activation purposes, depending on the desired applications. The main disadvantage of the magnetite versus the permanent magnetic powders is related to the lack of the control on the recovery time. Based on a recent paper, this disadvantage can be partially compensated by a bidirectional approach, as proposed by Tahmasebipour and Paknahad [3].

The disposal of a small amount of liquids is of increasing interest in the field of developing MEMS; certainly, micropumps and valves are critical components of these devices. Polymer magnetic films have been developed for over 20 years; based on the literature survey, these films are used in many applications, such as membrane actuators, magnetic micro-pumps, micro-mixer, micro-robots, micro-sensors, micro-concentrators, etc. [4,5,6,7,8,9,10,11,12,13,14,15]. These systems are based on several actuation principles such as: piezoelectric, electrostatic, thermopneumatic, electrochemical, bimetallic, shape memory alloy, and electromagnetic and their performances are gradually improved [5,16]. Starting from the initial films which assured limited displacements of few microns (usually below 10–20 µm) the actual films can assure tens of µm even at low currents [5,17,18]. 

The aim of this review is to highlight the most common magnetic polymer films used in developing microelectromechanical systems, from simple microvalves to micromixer, micropump, microsensors, drug delivery micro-systems, magnetic labeling and separation microsystems for specific medical purposes. Based on the literature survey, it can conclude that many MEMS require magnetic films; this is why, in the last decade, many devices were proposed, optimized and miniaturized. The advances assured by the modern technologies, including soft lithography, micro-molding or 3D printing, the design of these devices, and the flexible materials were able to assure the development of flexible/wearable devices, at relative low price, which can be attached to the human body, and which assure long term personalized therapy and/or monitoring based on the external control of the actuation. 

## 2. Common Polymers Used in Developing Magnetic Composite Films 

The most polymers used in the development of the polymeric films are: polydimethylsiloxane—PDMS; polymethylmethacrylate—PMMA, parylene or polyimides and the range of the polymers can be extended depending on the needs. Table 1 presents the main polymers used and their most important characteristics which recommend or limit their use in the development of the specific micro-devices.

The selection of the polymers is based on their overall performances but also on the final application and operational conditions. Depending on the final applications, more flexible or elastic materials are desired and from this point of view, the polymers mentioned in Table 1 cover the required range especially if consider also blends and layered structures. Considering, for instance, the Young modulus, it can conclude that depending on the final applications, elastic behavior can be considered from 0.36–0.87 GPa for PDMS and up to 3.8–12.2 GPa for polyimides. Most of the proposed polymers can be operated in aqueous media, at room temperature. Certainly, acidic or alkaline conditions, the presence of the specific solvents or the higher temperature can limit the use of certain polymers because the swelling rate, degradation or even dissolution can occur and alter the stability and the mechanical properties. Certainly, the mechanical parameter can be correlated with the actuation–displacement but, at the same time, with the energy consumption; it is worth mentioning that, in many medical applications, the energy saving is essential [19]. In addition, the young modulus is important because the flow rate of a micropump, the mixing capacity of a micromixer, etc., are proportional with the displacement of the magnetic films. Depending on the final application, the mechanical characteristics (and especially tensile strength) are important and depend not only on the nature of the material but also on the processing route. When discussing about the composite films, additionally, the content of the magnetic component, the surface chemistry of these particles, etc. are also important. For instance, PI/PDMS blends can be interfacially compatibilized by using reduced octadecylamine-functionalized graphene oxide [20] while, in the case of the soft PDMS-stiff epoxy resin, the compatibilization is assured by the use of 3-aminopropyltriethoxysilane [21]. In the case of layered membranes, the design of the properties can be done easily by properly combining the layers but the compatibilization is a real challenge, especially when layers are chemically incompatible. An extensive review is presented related to the PDMS bonding with other materials, including glass, silicon, PET, PVC, PS, PI or PMMA. In these cases, the treatment and compatibilization techniques are: corona-triggered surface activation, oxygen plasma surface activation, chemical gluing, and combined techniques [22].

PDMS-based membranes are extensively used for medical applications. Their manufacturing process present some specificities, among which include the thickness dependence of the mechanical properties [23] or the effect of the fringing field on the membrane curvature [24,25]. Usually, the thickness of the silicon magnetic membranes is between 25 and 200 µm but even magnetic membranes of 1–20µm are presented in the literature. The main advantage of using thinner membranes is correlated with the larger deflection but, fabrication issues occur [18,26]. Even if the devices are electrostatic, the works provide a design process able to make the membrane recover free from ghost solutions.

**Table 1:** Properties of different polymers used in the development of the microfluidic devices.

Polymer	Specific Properties	Ref. *
Polydimethylsiloxane (PDMS)	Ease of fabrication by rapid prototyping and good sealing, transparency in the UV-visible regions, low polarity, low electrical conductivity, and elasticity/flexibility; density of 970 kg/m^3^; Young’s modulus = 0.36–0.87 GPa, tensile or fracture strength is 3.5–7.65 MPa while elongation to break is 76%; Good chemical stability, being compatible with the following solvents: water, nitromethane, dimethyl sulfoxide, ethylene glycol, perfluorotributylamine, perfluorodecalin, acetonitrile, and propylene carbonate. Higher swelling ratios are reported for diisopropylamine, triethylamine, pentane, and xylenes (1.41–2.13 defined as follow: S = *D*/*D*_0_, where *D* is the length of PDMS in the solvent and *D*_0_ is the length of the dry PDMS). A major drawback of the PDMS-based materials is related to the high deformability. For instance, in the case of the microfluidic systems with thin wall, the diameter can be enlarged several times before failure.	[27,28,29,30]
Polymethyl methacrylate (PMMA)	Good transparency in the visible regions, but filters the UV light bellow 300 nm; durable density of 1180 kg/m^3^; the glass transition occurs between 100 and 130 °C; water absorption is 0.3%; Young Modulus is 2855 MPa, tensile or fracture strength is 70 MPa while the elongation to break is of 4.5%; PMMA is biocompatible and also biodegradable. Generally, PMMA is stable in most inorganic chemicals, aliphatic hydrocarbons, cycloaliphatic compounds, fats and oils at room temperature, and also to diluted acids and concentrated solutions of most alkalis at temperatures up to 60 °C but is attacked by chlorinated hydrocarbons, ketones, esters, ethers, alcohols and aromatic compounds.	[31]
Parylene (PAR)	Transparent material, the glass transition temperature <90 °C; depending on the composition, the melting point can vary between 290 and 420 °C; tensile strength is 45–69 MPa for Parylene N/C, the Young’s modulus is 2.4–3.0 GPa (N-C-F) while the elongation to break is 20–200% for Parylene C, can reach 250% for Parylene N while for Parylene F 10–50% at most. Good water absorption (<0.1%); good barrier properties in general; inert to most solvents up to 150 °C; parylene C became soluble in chloro-naphtalene at 175 °C while parylene N at 265 °C (solvent boiling point); diluted inorganic reagents (including acids, alkali, etc.) have no effect bellow 75 °C but, under severe conditions (concentrated acids, 75 °C for 30mins) swelling is observed (ranging from 0.7% for HCl to 8.2% for chromic acid); these polymers are biocompatible.	[32,33]
Polyimides (PI)	Strong dependence of the properties and performances of the polyimides can be correlated with the composition and synthesis/processing; aromatic polyimides are usually dielectric, tensile strength 72 MPa, their Young modulus can be 3.8–12.2 GPa while the elongation to break is only 8% depending on composition and processing, transparent material in the visible range (80–92% transmittance in the 420–900 nm); excellent thermal stability but also good chemical properties; The solubility is strongly dependent on the nature of the polyimides; there are some polyimides soluble in polar solvents (such as dimethyl acetamide, dimethyl formamide, N-methyl pyrolidone, m-cresol, as well as in conventional polar solvents such as tetrahydrofuran and chloroform) but other polyimides can be very stable. Loading polyimides with 0.5–1.4% carbon-based materials can greatly approve electrical properties; good biocompatibility and can act as an electrically triggering drug delivery support (if loaded with C).	[34,35,36,37]
Polyethylene terephthalate (PET)	Strong dependence of the properties and performances with the synthesis/ processing; Young Modulus is 2.8–3.17 GPa, tensile or fracture strength is 60–85 MPa while the elongation to break is of 20% but is also strongly dependent on the composition; transparent material in the visible range; good thermal stability, the melting point is 255–265 while the T_g_ is 67–140 °C; PET is insoluble in water, ethyl ether and most organic solvents but soluble in trifluoro acetic acid, DMSO, nitrobenzene, phenol and o-chlorophenol. The chemical stability in concentrated acids or alkali is poor, thus, limiting the use of this polymer.	[38]
Polyethersulphone (PES)	Polyethersulfone is a transparent material resistant to acids, alkalis, oils, greases, and aliphatic hydrocarbons and alcohols. It is attacked by ketones, ester, some halogenated and aromatic hydrocarbons, pyridine and aniline; it is very stable, being obtained up to 400 °C, being not oxidized up to 150–190 °C; the T_g_ is 190–290 °C; Young Modulus is 2.6 GPa, tensile or fracture strength is 83–85 MPa while the elongation to break is of 25–80% being strongly dependent on the composition and processing.	[39]
Polystyrene (PS)	Transparent, brittle, flammable, thermoplastic, stiff and hard material, obtained by polimerization of styrene. Polystyrene can be copolymerized or blended with other polymers, lending hardness and rigidity to many plastic materials. Flows when heated over 100 °C. Refractive index is 1.6. Soluble in benzene, toluene, ethylacetate, acetone, chloroform, trichloroethylene, cyclohexanone, MEK, THF, etc. Insoluble in water.	[40,41]
Polyaniline (PANI)	It is one of the best known, studied and applicable conducting polymer. Its properties strongly depend on the type of dopant and its concentration. PANI has many amine functional groups which interact with negative charge anion owing to its inherent cationic nature, such as easy chemical/electrochemical synthesis in a large scale, nontoxicity and good environmental stability.	[42,43]

* Some properties are extracted from: https://polymerdatabase.com (accessed on January 2022).

Usually, the increasing swelling ratio, as well as the solubilization or physico-chemical degradation behaviors are considered to be a limitation of the material. The chemical stability of these four polymers seems to be suitable for most applications in aqueous solutions, including in diluted acidic or alkaline solutions at room temperature for PDMS, PMMA and parylene. The swelling ratio is another factor that must be considered when choosing a polymer; certainly, lower swelling can assure a more predictable and constant behavior. However, the swelling behavior of the PDMS can be exploited to perform reaction within the microchanels or to modify the surface characteristics [27,44]. Koydemir and Kulah [45] evaluate the properties of Parylene C and PDMS and concluded that Parylene C is much more stable in most solvents, which makes it more suitable for microfluidic systems. Based on the mechanical properties of these polymers, PDMS and parylene have some advantages versus the other polymers, especially the much higher elongation to break.

Fluorinated polymers are increasingly used in various applications including drug delivery, tissue engineering, microfluidic and actuators, including artificial muscles. These polymers are especially recommended to applications because they are more restistant to chemicals and have a higher melting point and higher continuous-use temperature (up to 327 °C and 260 for PTFE) [46].

## 3. MEMS Type Devices Based on Magnetic Polymer Films

MEMS devices are, in fact, microsystems composed of micromechanical sensors, actuators, and microelectronic circuits. While technologies for manufacturing of microelectronic circuits are no longer a challenge, being a well-documented and experienced field over the years, current research in MEMS devices has been almost entirely engaged in the development of micromechanical sensors and actuators, known also as transducers [47].

During the last two decades, a wide range of MEMS were produced, most of them based on quite the same polymers and magnetic components, the major challenge being represented by their design [48,49,50,51,52,53,54,55]. Many approaches have been used in designing such devices, the biomimetic approach being also used in this field. For instance, Vignali et al. [50], designed and fabricated a 3D-printed pump mimicking the motion of the left ventricle using polyurethane (tensile strength: 0.8–1.5 MPa and elongation to break: 170–220%).

Polymers are found in various medical instruments with several biological and physicochemical characteristics that make them appropriate for their use in the biomedical field, whether we are discussing implantable devices or external devices. The flexibility of polymers is an essential feature that allows for the possibility to apply them in applications involving precise control of movable structures, such as MEMS actuators, with an emphasis on electromagnetic actuation [48].

A bidirectional magnetic polymer film actuator was proposed by Currie and Gray [48] based on a hybrid PMMA-magnetic polymer system that exhibited bidirectional deflection when exposed to a low magnetic field of 19–110 mT. The magnetic polymer actuator is composed by two circular diaphragms, one of 7 mm and a thickness of 0.2 mm and another of 2 mm diameter and 2 mm thickness, based on PDMS and permanent magnetic powder based on (Nd0.7 Ce0.3)10.5 Fe 83.9 B5.6, the average permanent magnetic field being of 25mT. The stroke volume of water is 0.5–6.8 uL at an applied magnetic field pf 19–110 mT; thus, coupling more such individual units, pumps, valves or mixers with adequate flow could be obtained. 

Polydimethylsiloxane films loaded with NdFeB powder can be prepared by mixing PDMS precursors (monomer—curing agent in a 10:1 ratio) with the magnetic powder. A special attention must be paid to the size and homogeneous distribution of the magnetic powder within the PDMS film. For instance, Iacovacci et al. [52] prepared the films by spin-coating (280 rpm/s acceleration ramps, 1400 rpm speed and 30 s overall spinning time) using 10, 30, 50 and 70% *w*/*w* NdFeB powder (prior milled under Argon to 5–10 µm). After the spin coating, a strong uniaxial magnet was used to obtain a preferential magnetization axis, followed by curing at 60 °C for 3 h. By using adequate electromagnetic fields (generated by two orthogonal pairs of Helmholtz coils and two pairs of Maxwell coils) steering and propulsion of the film could be assured. The as-obtained film could reach 11.5 mm/s, being proportional with the content of the NdFeB powder. By coating these films with multiple layers of polyelectrolytes (10 layers of chitosan/hyaluronic acid) the biocompatibility was well improved, the as-obtained film being much more adherent for the human cancer cell lines [52]. This research opens a new perspective in testing different drugs in cancer treatment but also in wireless transport of desired stem cells for therapeutic purposes. Lee et al. [51] proved that the magneto-active membranes actuator based on silicone and NdFeB microparticles could be optimized by an adequate curing procedure, the crosslinking degree and the alignment of the magnetic particles influencing the actuation capacity of the film. 

**Figure 1:**
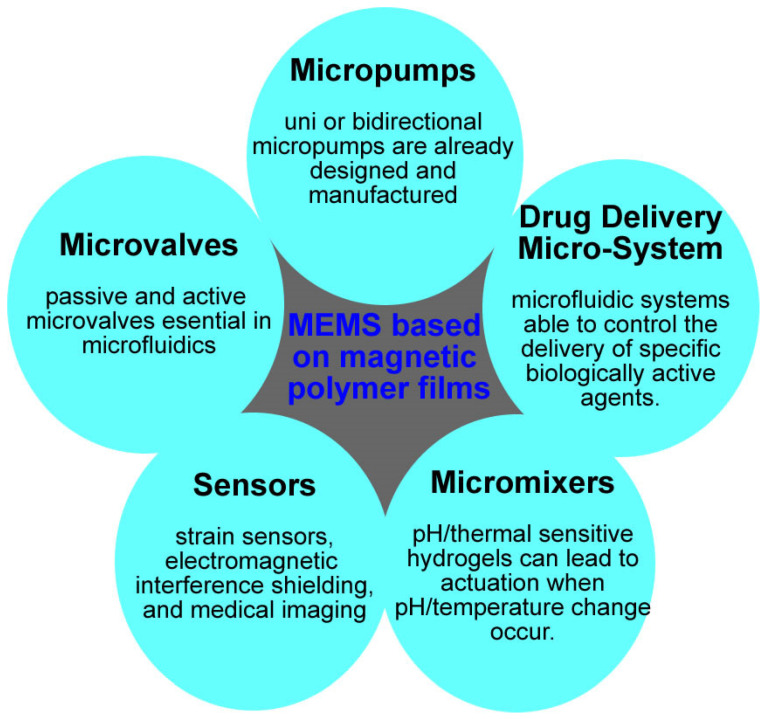
Main classes of MicroElectroMechanical Systems based on magnetic membranes.

The most used MEMS type devices containing magnetic polymer films are the following: microvalve, micropump, micromixer, microsensor, drug delivery systems, magnetic labeling and separation microsystems (Figure 1). Table 2 presents some of the most important characteristics and applications of the MEMS based on magnetic polymer films.

### 3.1. Microvalves

A microvalve is a key element in microfluidics that regulates the flow between two ports (inlet and outlet). Over time, microvalves have shown limitations in pumping rates at macroscopic level. Thus, different types of microvalves have been designed and manufactured to overcome those limitations. Currently, there are two main types of microvalves: passive microvalves and active microvalves. The active microvalves can be further categorized into mechanical and non-mechanical microvalves. Based on the initial state, the microvalves are divided in normally open, normally closed, and bistable microvalves [56,57]. The microvalve is widely used in many applications, such as life sciences (especially PCR amplification of the nucleic acids [58]), chemical engineering, microfluidics, printing devices, drug delivery, etc. 

Pramanik and Suzuki recently suggested the idea of a switchable microvalve fabricated by electropolymerized polypyrrole (Ppy) film doped with dodecylbenzensulfonate (DBS). Applying an appropriate potential successfully changed the state of the microvalve from almost hydrophobic to more hydrophilic. The contact angle for the as-deposited DBS-doped PPy films was 86° and decreased to 25° after applying a reductive potential of −0.8 V. This study provides a good solution for automating the transport of solutions, especially due to a higher level of integration in microfluidic devices [59]. Another example of a microvalve that was responsive to external inputs was fabricated from stimuli-responsive hydrogels. Eramo et al. used cross-linking, grafting and photopatterning of a poly(N-isopropylacrylamide) hydrogel to overcome limitations regarding design and costs for microfluidic devices fabrication. The suggested device had 7800 actuated micro-cages capable of isolating and releasing solutes with potential applicability in single cell manipulation and nuclear acid amplification test [60]. 

Nevertheless, magnetically actuated microvalves also offer a considerable number of advantages such as low power consumption, low operating voltage or simple operating principle [61]. A bidirectional electromagnetic microvalve for drug delivery application was proposed by Alam and Samad [62]. The proposed microvalve contained two arrays of equidistant micro-coils placed opposite to each other and a permanent magnet (NdFeB) attached to a flexible PDMS composite membrane. The magnetic force that displaced the PDMS membrane was 8.3 mN; the membrane displacement was found to be 400 µm. The interaction between coil and the permanent magnet generated a force on the membrane as high as 8.3 mN when the parameters were: 15 turns of coil, 200 mA of coil current, and 50 µm thickness of PDMS membrane.

### 3.2. Micromixers

Technological processes that take place at the macroscopic level often require mixing operations for various fields of interest from combustion engines or reactors to pharmaceutical and cosmetic formulations. This mixing operation had also become of interest in the context of the emergence of microfluidics at the microscopic scale [63]. Unfortunately, even if, from a mathematical point of view, diffusion is expected to be faster in the microscale, limitations occur due to low a Reynolds number, gives a laminar flow to the microscale system, slowing down the mixing capacity of two fluids in such a system [64]. Hence, microfluidics also focused on the idea of designing proper devices in order to enhance mixing performances. Micromixers are classified into two general types: passive and active. Passive micromixers are defined as devices that do not use any external stimuli to control the mixing process and that rely mainly on diffusion processes; mixing efficiencies may be enhanced by tuning microchannels in different types of geometries. In most studies based on this hypothesis, microchannels have been designed in the form of serpentines, by applying various geometries such as split-and-recombination channels (SAR) [65], Koch fractal [66] or mathematical equations whose graphic representations have curved geometries that can induce Dean vortices and, thus, can potentiate the mixing of the fluids [67]. Yin et al. proposed a 3D microfluidic device with superior mixing performances. The study consisted in designing and manufacturing a microchannel-based device with geometries inspired by mathematical functions such as Archimedean, Fermat and hyperbolic spirals. The study combined numerical simulation of mixing efficiency with visualization experiments to demonstrate the feasibility of using it as an integrated point-of-care (PoC) device. For a flow characterized by a Reynolds number of 5, the mixing index increased with decreasing channel width, the best performance being for Fermat’s spiral geometry, with a 0.2 mm width channel, experimentally reaching 99% mixing efficiency. Fermat’s spiral was integrated into a 3D microfluidic device that tracked the detection of cardiac troponin I (cTnI) to diagnose acute myocardial infarction (AMI). Detection time of only 10 min, correlated with a linear response (R^2^ = 0.99) for the working range 30–1920 pg/mL, and very low detection limits (30 pg/mL) demonstrates the feasibility of using this device for PoC applications [67]. Similar, high mixing efficiencies can be reached by combining SAR channel with primary Koch fractal (97.12%) or secondary Koch fractal (97.44%) [68]. A vacuum pouch microfluidic (VPM) mixer was manufactured from a polypropylene vacuum pouch and a thin-film micromixer based on curved microchannel for on-site detection of heavy metal ions using a colorimetric detection method. The VPM micromixer was designed by embedding a thin-film micromixer with curved microchannel in a polypropylene vacuum pouch. After piercing the vacuum pouch, fluids (reagents) are absorbed into the micromixer due to pressure difference between the atmospheric pressure and the negative pressure released from the previous vacuum system, initiating the mixing process [69]. PDMS thin film patterned with microchannels rolled around a core, using plasma bonding, formed an out-of-plane (3D) single- and double-spiral microchannel with very good performances for heat sink and heat exchanging applications [70]. 

Active micromixers on the other part possess some external energy sources that can disturb the fluids in the system boosting the mixing effect. These external sources may be electrical or magnetic, and from pressure or sound, and can generally assess higher mixing efficiencies combined with a more controlled and rapid process than passive micromixers [71,72]. 

Autonomous micromixers can be achieved by using proper microfluidic systems [8]. The actuation is assured by the use of different Ni blade designs and pH-sensitive hydrogels geometries and dimensions. Because of the pH variation, these hydrogels change their characteristics; they can shrink (for instance, if poly(AA-HEMA) is used, shrinking occurs in acidic conditions) and expand (if the pH is raised above the transition point). In a similar way, temperature-sensitive mixers can be designed; in this case, the actuation is generated because of the changes that occurs as a consequence of the temperature variation. The microfluidic system is externally controlled by using an external magnetic stirrer, but ongoing research is in progress to develop microfluidic systems with integrated three-phase micromotors to remove the need of an external magnetic stirring unit. 

Rahbar, M., et al. proposed an active micromixer with a cilium geometry fabricated from a PDMS composite doped with a magnetic powder with a grain size of 5–10 µm with potential use in µTAS and lab-on-chip devices. The magnetic polymers cilia showed better mixing efficiencies compared to the inherent process of diffusion that took place when two or more substances were put together in a system. Mixing experiments showed that applying a magnetic field of 7 mT and 60 Hz frequency led to almost 100% mixing efficiency for a cilium of 1.5 ± 0.01 mm length and 130 ± 5 µm diameter. The complete mixture took approximately 15 min. This time can be shortened by increasing the number of cilia used. For example, under the same experimental conditions of 7 mT and 60 Hz magnetic field applied, by using an eight cilia system, 85% mixing efficiency was achieved in 70 s, while, by using a single cilium, this percentage was reached in 3.5 min [72]. 

For glucose monitoring, Pawinanto et al. [73] proposed an electromagnetically driven active microfluidic mixer with micropillar on a polymer-based flexible membrane. The electromagnetic actuation of the membrane is intended to increase the vortices inside the mixer chamber, which is anticipated to improve the mixing index and to shorten the mixing time, which results in significantly better mixing properties than those previously reported. The structures are constructed of the polymer PDMS and were created using the soft lithography technique, which is a simple and cost-effective method.

### 3.3. Microsensors

Microsensors are another branch of microfluidics that have received special attention, primarily due to the progress in the use of elastic polymers that are easy to manufacture and that are sensitive to physiological variations. However, the design and integration of microsensors is currently a limitation, especially in terms of sensitivity, response time and working range. In an attempt to overcome these limitations, research focused on using different types of polymers, in particular to lower the detection limits of devices. An electrochemical impedance spectroscopy (EIS) microsensor, for example, was used to dose traces of glyphosate in water samples, after molecularly imprinted chitosan was covalently attached on the surface of a microelectrode previously treated with 4-aminophenylacetic acid (CMA). The CS-MIPs/CMA/Au microsensor’s EIS responses to glyphosate were well-proportional to the concentration in the range of 0.31 × 10^−9^ to 50 × 10^−6^ mg/mL and a detection limit of 1 fg/mL. This microsensor also demonstrated strong reproducibility and repeatability, as well as great selectivity, and may be utilized to detect glyphosate in river water [74]. According to [75], sensor sensitivity to normal stress may be adjusted, for example, based on the form ratio of PDMS. The capacitance change reached up to 37% under a 10 N load for a 9 mm^2^ sensor built with a specific PDMS film when the optimum form ratio was used in the sensor design. Moreover, sensors based on piezoelectric resonators with sensitive sheets of elastic polymers were used to measure the concentration of chemicals in gases and liquids.

A strain sensor based on electromagnetic functionalized polyaniline/polyurethane acrylate/Fe_3_O_4_ (PANI/PUA/Fe_3_O_4_) micro-ribbons was reported. The conductivity of the PANI/ PUA/ Fe_3_O_4_ composite was 2.04 × 10^−3^ S·cm^−1^. The strain sensor based on PANI/PUA/Fe_3_O_4_ micro-ropes showed a linear response to the applied strain from 0% to 199.9%, as well as a quick and repeatable response to pressure and finger motion. The electromagnetic functionalized ribbons exhibited superparamagnetic behavior at 300 K. The electromagnetic functionalized ribbons are potentially convenient for various applications, such as strain sensors, electromagnetic interference shielding, and medical imaging, etc. [76,77].

Magnetic functionalities can provide a sense of displacement, orientation or proximity for the recent field of stretchable electronics. Soft giant magneto-resistive elements exhibiting the same sensing performance as on conventional rigid supports, but with fully strain invariant properties up to 270% stretching, has been demonstrated. With their unique mechanical properties, these sensor elements readily conform to ubiquitous objects of arbitrary shapes, including the human skin. Stretchable magneto-electronic sensors can equip soft and epidermal electronic systems with navigation, orientation, motion tracking and touchless control capabilities. A variety of novel technologies, such as electronic skins, smart textiles, soft robotics and actuators, active medical implants and soft consumer electronics will benefit from these new magnetic functionalities [78].

### 3.4. Magnetic Labeling and Separation

Several label-free biosensing systems based on liquid crystals (LCs) have been developed during the last decade. These include biodetection at the LC–glass interface, typically comprising a thin film of LC sandwiched between two glass substrates to produce an LC cell, and at the LC–aqueous interface in the form of LC films or LC-in-water droplets. Although the biosensing capability of both thermotropic and lyotropic LCs has been proven, most LC-based biosensors described to date have used the nematic 5CB as the sensing mesogen. LC–photopolymer composites have demonstrated important implications in biomedical applications, such as label-free and single-substrate biodetection. Lee et al. [79] incorporated a photocurable prepolymer (NOA65) in a nematic LC E7 or AY40-006 one. This mixture was spin-coated on a glass substrate treated with dimethyloctadecyl[3-trimethoxysilyl)propyl] ammonium chloride (DMOAP) and then irradiated with ultraviolet (UV) light. The accumulated and polymerized NOA65 at the LC–glass interface weakened the anchoring strength of DMOAP during the photopolymerization process, resulting in a decrease in the pretilt angle of LC and allowing the LC molecules to be more easily disturbed in the presence of biomolecules when compared to vertically aligned LC in the absence of polymerized NOA65. As a result, NOA65 incorporated within the LC film provided a signal amplification. When the LC–photopolymer composite film containing AY40-006 and 4% (%wt) NOA65 was exposed to UV at 15 mW/cm^2^ for 30 s and was used as the biosensing mesogen, the limits of detection were 1.6 × 10^−12^ g/mL for bovine serum albumin (BSA) detection and 2.1 × 10^−8^ g/mL for the immunoassay of the CA125 cancer biomarker CA125, both limits of detection being significantly lower than those un-irradiated with UV light.

For the manipulation and separation of single or bulk amounts of cells, different microfluidic technologies have been developed, based on flow cytometry, photophoresis, acoustophoresis, dielectrophoresis, and magnetophoresis. Magnetophoresis, in comparison to other methods, has distinct advantages due to the use of magnetic fields, including remote noninvasive manipulation, programmability, lower heat generation, and biocompatibility, as well as the ability to fix cells during analysis and to adjust the distance between cells [80]. In addition, magnetically labeling the cells can be done preferentially and, in this case, their separation can be of great interest in biological applications and medicine. Starting from this aim, there are several papers in the literature that have dealt with manipulation of these cells, including in microfluidic devices. Faivre et al. [81], for instance, proposed a PDMS-based film loaded with iron carbonyl that was able to capture/deviate the magnetically labeled cells or superparamagnetic beads when it was passing in a plexiglass-based microfluidic system that had a channel diameter of 1mm and that contained 2 permanent magnets (25 × 10 × 5 mm^3^ and 1.2 T at the pole and polarization in the longest dimension).

### 3.5. Micropumps

A bidirectional approach using a cylindrical device using two discs, each holding a permanent magnet, was presented by Amrani et al. [61]. This micropump was based on a specific axial displacement of the two magnet-discs, which could be operated with a flow rate of 37 mL/min, with a back pressure of 1.98 kPa and which allowed the change of the flow according to the applied currents. The first micropump based on PDMS and Fe_3_O_4_ was reported by Tahmasebipour and Paknahad, in 2019 [3]. The major advance reported in this paper is related to a bidirectional mechanism generated by a secondary coil situated above the magnetic film. By the alternative switching ON/OFF of the two coils, the magnetic film will return to the initial state, not only due to the elasticity of the film but also due to the magnetic force generated by the second coil. The functionality of the micropump was assured by the applied magnetic field (3.28 mT) which was generated by the two coils’ 15 turns each, with a consumption of 1.1 W/coil. Under these conditions, a displacement of 24 µm in 10 s was obtained for the composite Fe_3_O_4_/PDMS film containing 5% magnetite. The proposed bidirectional micropump could assure a flow of 1.25 µL/min whereas the unidirectional micropump could assure 0.93 µL/min. 

A recent paper published by Suna et al. [82] compared the influence of the nature of the magnetic particles incorporated in PDMS films. Three magnetic particles were considered: lauric acid coated superparamagnetic iron-oxide nanoparticles, lauric acid coated carbonyl iron microparticles and pristine carbonyl iron microparticles; they concluded that the pristine carbonyl iron-based membrane had the largest deflection value of 762 µm at a field of 0.27 mT for the films of 6.2 mm in diameter. 

Silicon rubber-based films loaded with NbFeB can be used in order to develop micropumps with pumping and mixing capabilities in order to assure fast and ultrafast response, with potential applications in developing medical devices and soft robotics [83]. Silicon rubber was also loaded with atomized iron powder in order to develop magnetic polymer composite films for developing transdermal drug delivery systems. Jayaneththi et al. [53] designed and realized a wireless pumping system able to provide the drug according to a pulsatile, zero-order delivery profile through a microneedle [40]. The device was designed as a pneumatic-hydraulic actuation system that was able to provide a flow rate of 9.07 ± 0.28 to 108 ± 2.6 µL/min.

### 3.6. Drug Delivery Micro-Systems

A special class of micropump was represented by the drug delivery micro-systems, which were especially designed for the treatment of specific diseases, as presented below. 

**Figure 2:**
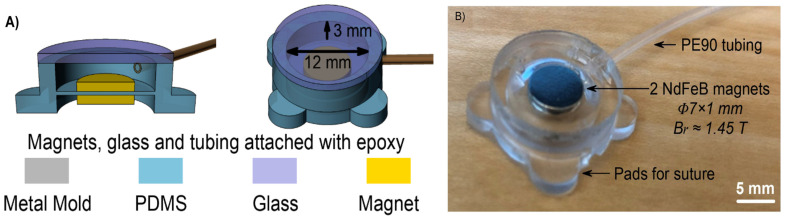
(**A**). Schematic views of the magnetic microfluidic pump and (**B**). implantable magnetic micropump (reproduced from [54]).

An implantable magnetic micropump for in vivo bone remodeling was also reported by Chen et al. [54] (Figure 2). They designed a microfluidic pump of 22 mm diameter and 5 mm in thickness using 2 NdFeB magnets in PDMS according to the schematic representation from Figure 2A. The actuation was assured externally, by using an actuator and a larger magnet. The in vivo test was done on a Fisher-344 rat; the micropump was connected to the intramedullary cavity of the femur by a tube. A back pressure of 38 mmHg in the intramedullary cavity was generated and created a basis for treatment of osteoporosis, for instance. 

Microfluidic systems based on PDMS film loaded with carbonyl iron as an actuator were designed, realized and further used to deliver ciprofloxacin against *Escherichia coli*. The antibacterial activity was dependent on the release characteristics, promising results being obtained, which could probably be translated into developing clinically relevant devices [77].

Another implantable, wireless device with potential medical application was published by Lee et al. [84] and can be of real benefit for the patients with diabetes. There is a lot of research in developing drug delivery systems for insulin [85,86], including a wide range of implantable microdevices; however, many of them require electrical power supply and electronic circuit components; thus, their implantation is limited. Because of these limitations, a magnetically driven micropump was proposed. This pump comprised a drug reservoir, and an actuator that was externally activated by a proper magnetic field. The in vivo study proved that the dosage of insulin was controlled by the number of actuation. 

A thin magnetic micropump embedded in a contact lens that is capable of on-demand one-directional drug delivery was reported. The proposed micropump can be actuated by the external magnetic field whenever needed, without the need of a battery. A micro check valve was integrated with the micropump for one-directional drug delivery from the micropump to the post-lens tear film. With actuation of the external magnetic field, the micro check valve was opened, and on-demand drug release can be realized. On the contrary, without an external magnetic field, the micro check valve is closed, and the undesired drug release can be prevented. Through the control of the strength and the frequency of the magnetic field pulse, on-demand drug release and controlled dose can be achieved [87].

**Table 2:** Microelectromechanical systems based on magnetic polymer films.

Crt. No.	Film	Composition	Applications	Reference
1	PDMS-(Nd-Ce)FeB	Magnetic composite polymers based on PDMS and hard rare earth magnetic powder, (Nd_0.7_Ce_0.3_)_10.5_Fe_83.9_B_5.6_ at a ratio of 20:80 (wt)	Delivery, mixer	[48]
2	Silicon rubber-NbFeB	Silicon rubber loaded with NbFeB	Transport and mixing	[83]
3	PDMS-NdFeB-polyelectrolytes	PDMS membranes and 10%, 30%, 50% or 70%) NdFeB powder were obtained, proving the possibility of using these films as film-shaped microrobots.	Delivery and Transport	[52]
4	shape memory polymer/polyimide laminate actuator	Shape memory polymer/polyimide laminate actuator, wirelessly activated by external RF electromagnetic field at specific frequency of heater. Cu-clad PI.	Drug Delivery	[55]
5	Fe_3_O_4_/PDMS	PDMS-based composite film loaded with 5% Fe_3_O_4_ is used in a bidirectional approach using two identical coils with 15 turns and operated alternatively.	Micropump, Drug Delivery	[3]
6	Silicone rubber-Fe	Silicon rubber membranes loaded with 30% (*w*/*w*) atomized iron powder.	Transdermal Delivery	[53]
7	PDMS-NdFeB	NdFeB magnets were entrapped into PDMS film	Intramedullary delivery	[54]
8	Stimuli responsive polymer—Ni	Different stimuli (pH and temperature) responsive hydrogels are patterned at the center axis of the electroplated Ni rotor. By changing the conditions, these hydrogel rings shrink expansion and, thus, assure, within a microfluidic chamber, a mixing process.	Sample Preparation	[8]
9	PDMS-Fe(CO)_5_	PDMS-based film is loaded with iron carbonyl and used for the manipulation (capture) of the magnetically labeled cells.	Cell and Particle Manipulator Chemical Release of Antibiotics	[77,81]
10	Polystyrene-Fe_3_O_4_	Magnetic Fe_3_O_4_ nanoparticles were encapsulated by polystyrene.	Microelectromechanical systems (MEMS)	[78]
11	PANI-Fe_2_O_3_	Magnetic nanoparticles are coated with PANI	Signal transducer for application in a disposable membrane strip biosensor.	[88]

## 4. Design and Fabrication of the Microfluidic Systems

**Figure 3:**
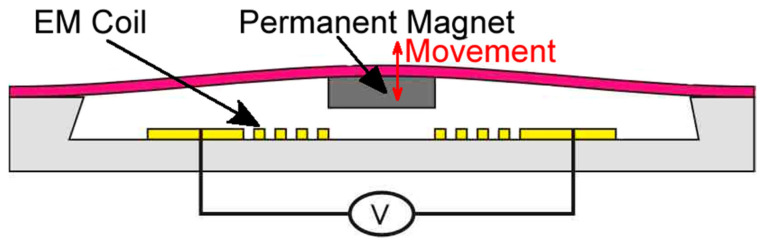
Working principle of the electromagnetically induced actuation, adapted according to [18].

The functionality of the microfluidic systems is strongly associated with the design and nature of the active and passive components; however, the influence of the microscale must also be considered. Mechanical and non-mechanical actuation can be used to generate movement. It is generally accepted that mechanical actuation is more advantageous in term of controllability, high vibration rate and large membrane deformation [18]. The polymer magnetic films described above are fit to be used in electromagnetically actuated MEMs, the schematic representation being highlighted in Figure 3. In this approach, the polymer film bears the magnetic material, while the coils are embedded or attached to the stationary part of the MEMs. Oppositely, the coils can be attached on or embedded into the movable film while the magnetic component is attached to the stationary part. Based on the design, the membrane deformation can lead to specific applications such as micromotors, micropumps, micromixers, or magnetorestrictive devices. 

**Figure 4:**
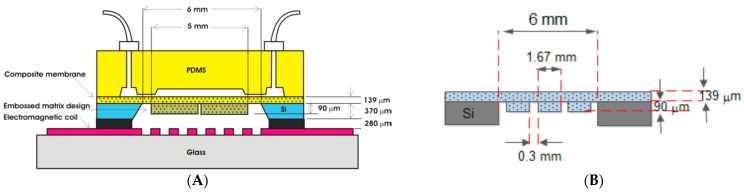
(**A**) The design of the proposed EM micropump and (**B**) the matrix-patterned magnetic array structure [57].

Instead of a permanent magnet, as presented in Figure 2, a matrix-patterned magnetic array structure can be used as presented by Said et al. [89] (Figure 4). Based on their study, the PDMS—patterned magnetic array containing 6% NdFeB, was able to generate a 12.87 µm deflection and a flow rate of 6.523 nL/min when a 3 × 3 matrix-patterned magnetic array structure is used while 10 Vpp square wave, 0.235 mA and 1 Hz frequency was applied to the micro-coil (generating 0.63 mT).

**Figure 5:**
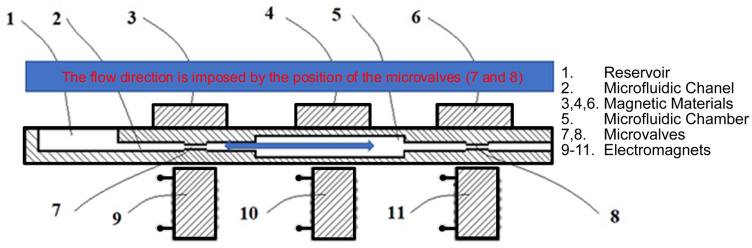
Schematic representation of a micropump actuated by 3 electromagnets, one microfluidic chamber and two restrictors able to generate a specific flow direction.

The microfluidic chamber, as presented in Figure 5, can work alone or additional microvalves can be considered to generate directional flow, as we highlighted in a patent application [90]. The micropump can act as a drug delivery system; in this case, the reservoir is filled with the drug solution, the microvalve (7) is locked, the microfluidic chamber is compressed by activating the electromagnet 10, followed by the closing of the microvalve 8. In this way, the chamber 5 is prepared to be filled with liquid when the microvalve 7 is open and the electromagnet 10 is released. To deliver the liquid from the microchamber, the microvalve 7 is closed again and the microvalve 8 is released and, again, the electromagnet 10 is activated to force the liquid out. In a repetitive manner, the device can be used as a drug delivery device. It is also important to mention that the simple device can also be operated in an opposite flow; in this case, the microfluidic device can be used for sampling liquids. In this case, the actuation sequence of the three electromagnets will be adjusted to move the liquid to the reservoir and, in this case, detachable reservoirs can be used to be able to get individual samples, in time. In both cases the reservoir can have a sense valve allowing to equalize the pressures while in the case of the using this system as a sampling device, the reservoir can even be vacuumed in order to assist the sampling.

The final application, as well as the performances of the derived MEMs, were proportional with the design and magnetic force and the deformation as described by Yunas et al. [18]. Depending on the desired application, the active/mobile component could be membrane but also pillars or cilia (especially for the development of micromixers). These 3D structures are usually obtained by soft lithography, micro-molding or 3D printing techniques.

The use of the soft lithography can be used in developing the complex systems and can be conducted in several steps, starting with the development of the electromagnetic part, followed by the fabrication of the magnet-o mechanical part, and, finally, by attaching the two components using adequate epoxy resins.

3-D printing is an alternative way of generating even complex 3D-structures. Various printing approaches can be used, such as: stereolithography- SLA [91], inkjet (including multijet) [92,93] or fused deposition modeling (FDM) [94,95] techniques. Among these, stereolithography offers some specific advantages versus the other printing techniques, especially better resolution, tighter tolerances and better compatibility with the thermoset polymers [96]. Much higher resolution could be achieved by two-photon photolithography-2PP down to 200 nm; however, this technique is very slow and it is usually used to print cilia-like structures. Unfortunately, the resolution of the SLA technique on PDMS was better than 330; however, no high resolution could be achieved by using commercially available devices. SLA can extend its functionality down to 2 × 2 × 1 um resolution if adequate digital micromirrors are used [96].

Microfluidic pumps for wearable biomedical applications were firstly proposed in 2009 and, since then, have been continuously improved [95,97,98,99]. Such devices can be used in a predefined manner and can assure a long term delivery of the active agents in a programmable (variable) dosage, over time. For instance, Fiering et al. [97] proved the concept at the in vivo level by dosing a glutamate receptor antagonist to the cohlea of a guinea pig, for 3 months. In 2015, Thomas et al. [95] developed a wearable microfluidic pump by 3D printing using PLA as base and a 750 µm thick Fe-PDMS polymer film containing 30%, 40% or 50% Fe. The best performance was obtained using the Fe-PDMS film containing 40% Fe and a flow rate 2.2–2.4 µL/min was achieved, depending on the frequency. Recently, Zhao et al. [98] realized a nozzle-diffuser microfluidic pump based on poly (vinylidene fluoride-trifluoroethylene) (P(VDF-TrFE)) (an ferroelectric polymer) loaded with 0, 0.3%, 0.7% and 1.1% Al_2_O_3_@CNT core shell structure. This micropump can assure a controllable flow rate between 13 and 135 µL/min being safe and bendable so can be easily attached to a human arm, can be operated at 50 V.

Certainly, additional techniques such as electrospinning can be used to develop films with oriented or un-oriented patterns to assure specific functions within the more complex devices, including advance separation and cell growth and manipulation under biomimetic conditions [93,100]. Interconnection of the individual units in microfluidic multi-organ-o-a-chip and the possibility of assuring crosstalk of these tissues from different chambers can be also assured by using 3D-printing [101].

Porous foams were also integrated into the actuated film, and in this way, a reciprocation displacement pump was developed using a nozzle diffuser approach. The open-porous foam membrane is used in order to simultaneously assure filtration and pumping. This approach can be used in microfiltration being less sensitive to the fouling due to the intrinsic vibration of the membrane [102].

## 5. Conclusions

Composite films based on several polymers and magnetic powder are increasingly developed for the development of the microfluidic systems; they include microvalves, micropumps, micromixers, microsensors, drug delivery micro-systems, magnetic labeling and separation microsystems, etc. Poly(dimethylsiloxane) and Parylene C/N seem to be suitable polymers having adequate stability in a wide range of solutions, including aqueous solution at moderate acidic or alkaline pH. Moreover, the mechanical properties including elongation to break and tensile strength are proper for the most applications. From the point of view of the preparation/processing and price, PDMS is most convenient and this is why PDMS-based films are more frequent comparing to the other polymers. To make the PDMS easily to be removed from the mould, a physical (to grease the mould with silicon oil) or a chemical approach (by functionalizing it with adequate silanisation agent) can be used. If superior properties are requested, PDMS-based materials coated with parylene, or even pure parylene films, can be used, thus, taking – about the compatibilization of the layers by using adequate techniques.

## 6. Challenges and Future Perspectives

The magnetic films are increasingly used in developing microelectromagnetic systems with increasing performances. These devices can be used as valves, micropumps, robots, manipulators, sample preparation and mixing, etc., their performances being dependent on the nature and ratio of the polymer: Magnetic powder, on processing and design. Further improvements are expected; some recent works have highlighted the potential improvements of the design but also on the nature, shape, size and composition related optimization of the magnetic powders. It is also worth to mention that these microfluidic systems are improving from many point of view, they were continuously miniaturized and their properties can recommend them for a wide range of applications, including biomedical field. Moreover, these devices are currently adapted to be used as wearable devices and, thus, to assure personalized treatment and/or diagnosis because the actuation can be externally controlled and even variable actuation profile can be assured.
